# Mortality in the Medicare Population and Chronic Exposure to Fine Particulate Air Pollution in Urban Centers (2000–2005)

**DOI:** 10.1289/ehp.11449

**Published:** 2008-08-12

**Authors:** Scott L. Zeger, Francesca Dominici, Aidan McDermott, Jonathan M. Samet

**Affiliations:** 1 Department of Biostatistics; 2 Department of Epidemiology, Johns Hopkins Bloomberg School of Public Health, Baltimore, Maryland, USA

**Keywords:** ecologic bias, fine particulate matter (PM_2.5_), heterogeneity, log-linear models, Medicare, mortality, prospective studies

## Abstract

**Background:**

Prospective cohort studies constitute the major source of evidence about the mortality effects of chronic exposure to particulate air pollution. Additional studies are needed to provide evidence on the health effects of chronic exposure to particulate matter ≤ 2.5 μm in aerodynamic diameter (PM_2.5_) because few studies have been carried out and the cohorts have not been representative.

**Objectives:**

This study was designed to estimate the relative risk of death associated with long-term exposure to PM_2.5_ by region and age groups in a U.S. population of elderly, for the period 2000–2005.

**Methods:**

By linking PM_2.5_ monitoring data to the Medicare billing claims by ZIP code of residence of the enrollees, we have developed a new retrospective cohort study, the Medicare Cohort Air Pollution Study. The study population comprises 13.2 million participants living in 4,568 ZIP codes having centroids within 6 miles of a PM_2.5_ monitor. We estimated relative risks adjusted by socioeconomic status and smoking by fitting log-linear regression models.

**Results:**

In the eastern and central regions, a 10-μg/m^3^ increase in 6-year average of PM_2.5_ is associated with 6.8% [95% confidence interval (CI), 4.9–8.7%] and 13.2% (95% CI, 9.5–16.9) increases in mortality, respectively. We found no evidence of an association in the western region or for persons ≥ 85 years of age.

**Conclusions:**

We established a cohort of Medicare participants for investigating air pollution and mortality on longer-term time frames. Chronic exposure to PM_2.5_ was associated with mortality in the eastern and central regions, but not in the western United States.

Particulate matter (PM) air pollution is a global public health problem (Cohen et al. 2004). In developing countries, levels of airborne PM still reach concentrations at which serious health consequences are well documented (Chhabra et al. 2001; Ostro et al. 1999a, 1999b; Vichit-Vadakan et al. 2001). In developed countries, recent epidemiologic studies show evidence of continued adverse effects, even though PM levels have declined in the last two decades (Dominici et al. 2006; Jerrett et al. 2005; Laden et al. 2006; Pope et al. 2002). Increased mortality associated with higher levels of PM air pollution has been of particular concern, giving an imperative for stronger protective regulations (Bachmann 2007; Samet et al. 2006).

The evidence on PM and health shows acute and chronic effects (Pope and Dockery 2006). The London Fog of 1952 provides dramatic evidence of the risk of extremely high levels of PM air pollution over a period of about a week (Bell and Davis 2001; Bell et al. 2004; Logan 1953). Multisite time-series studies estimate associations between the risk of death and the level of air pollution shortly before death (shorter-term effects). These studies have provided evidence that far lower levels of PM than those that occur during events like the London Fog are still associated with increased risk over several days (Dominici et al. 2006, 2007; Katsouyanni et al. 1997; Lee et al. 2000; Samoli et al. 2001). Cohort studies estimate associations between time to death and exposure to air pollution over multiple years (longer-term effects). The design of these studies involves follow-up of cohorts for mortality over periods of years to decades and an assessment of mortality risk in association with estimated longer-term exposure to air pollution (Dockery et al. 1993; Hoek et al. 2002; Jerrett et al. 2005; Krewski et al. 2004; Laden et al. 2006; Pope et al. 1995, 2002). The exposure indicator in these studies was long-term average air pollution concentration, and time-varying exposures were not used, except in the most recent updates of several cohorts (Laden et al. 2006; Pope et al. 2002). Hence, inferences about the relative risks of chronic exposure derive from comparisons across study cohorts in geographic units with differing long-term PM levels.

Künzli et al. (2001) have reviewed and compared time-series studies and cohort studies. They point out that air pollution might increase *a*) the risk of underlying diseases leading to frailty and the shorter-term risk of death among frail persons, *b*) the risk of chronic diseases leading to frailty but without relation to timing of death, and *c*) the shorter-term risk of death among frail persons but unrelated to risk of chronic diseases. They note that time-series studies capture items *a* and *c* but do not provide any information on item *b*; can be affected by confounding bias due to lack of control of time-varying covariates; and are useful to establish causation and to assess relative magnitude of effects across subgroups. On the other hand, Künzli et al. (2001) note that cohort studies capture items *a* and *b* but, in the absence of time-varying exposure, provide very little information on item *c*; can be affected by ecologic bias due to comparison of mortality risks across heterogeneous groups; and can be used to estimate years of life lost.

Because of their complexity and costs, only a small number of cohort studies have been conducted. The most rigorously executed, including the Harvard Six Cities Study and the American Cancer Society’s Cancer Prevention Study II (CPS-II), have provided generally consistent evidence for an association between average exposure to PM air pollution over a decade and increased all-cause and cardiorespiratory mortality (Dockery et al. 1993; Laden et al. 2006; Pope et al. 1995, 2002). Both studies compared mortality rates across counties or larger geographic units with different long-term PM levels to estimate relative risks. The results of these studies, rather than of the time-series studies, have been used to quantify the risks of PM exposure for consideration of alternative values for the U.S. National Ambient Air Quality Standard for PM [U.S. Environmental Protection Agency (EPA) 2003]. These results have also been used to estimate the global burden of disease attributable to air pollution (Cohen et al. 2005).

Additional cohort studies are needed to confirm associations between multiyear average exposure to PM and mortality, to broaden the populations studied, to reduce the degree of geographic averaging of the exposure measure—a source of ecologic bias—and to refine the estimates by regions, age, and socioeconomic status (SES) categories across which PM exposures may vary. Toward this end, we have used data from the U.S. Medicare system, which covers nearly all persons ≥ 65 years of age in the United States. We linked Medicare mortality data to the PM_2.5_ (PM ≤ 2.5 μm in aerodynamic diameter) air pollution monitoring data to create a new retrospective cohort study, the Medicare Cohort Air Pollution Study (MCAPS), a study population of 13.2 million persons residing in 4,568 ZIP codes in urban areas having geographic centroids within 6 miles of a PM_2.5_ monitor. We have previously described this general approach and the comparability of risk estimates based on MCAPS with estimates from the Harvard Six Cities Study and CPS-II (Eftim et al. 2008). In this article, we report on the relationship between 6-year average exposure to PM_2.5_ and mortality risk in the MCAPS over the period 2000–2005. Our objective is to provide new evidence about the relative risk of death associated with chronic exposure to urban PM_2.5_ by region and age-defined subgroups.

## Materials and Methods

MCAPS is a retrospective study of a cohort of 13.2 million persons ≥ 65 years of age enrolled in the U.S. Medicare system during the 6-year period 2000–2005. To create the cohort, we used the Medicare enrollment file for the study period, which provides a listing of all Medicare enrollees, along with demographic information (age, race, and sex) and ZIP code of residence. New participants enter each year as they enroll in Medicare, making this a “dynamic cohort.”

More specifically, the cohort consists of all those ≥ 65 years of age who enrolled in Medicare between 2000 and 2005 with ZIP code centroids within 6 miles of a U.S. EPA PM_2.5_ monitoring station. Although the Social Security Administration maintains the addresses of those enrolled in Medicare, the Center for Medicaid and Medicare (CMS) provides an annual report of Medicare enrollees by ZIP code (often referred to as the enrollee file). Medicare enrollees enter the cohort on reaching their 65th birthday or on 1 January 1999 should they be ≥ 65 on that date. A small number of individuals enroll in Medicare the year after their 65th birthday, and those individuals enter the cohort on January 1 of the year of their enrollment. Individuals contribute time to the cohort until they die or are otherwise censored. Censorship occurs when individuals move to a ZIP code > 6 miles from a U.S. EPA PM_2.5_ monitoring station or are no longer reported in the enrollee file. We calculated age-specific mortality rates as the total number of deaths occurring within an age group and ZIP code divided by the total person-years contributed by that age group and ZIP code.

We obtained the date of death from the CMS. The date of death is provided to CMS by the Social Security Administration, rather than by the National Center for Health Statistics (NCHS), which maintains the national death certificate system. To validate the mortality data from the CMS, we compared annual age- and sex-adjusted mortality rates from the CMS with the corresponding rates calculated from NCHS data for the 250 largest counties for the year 2000. The correlation coefficient was 0.998, indicating a high level of agreement between the two sources of mortality data aggregated to the county level—the finest partition available from the NCHS—for the 1-year period.

For this article, the outcome measure is the 6-year (2000–2005) mortality rate for persons residing within each of 4,568 ZIP codes for each of three age strata: 65–74, 75–84, and ≥ 85 years of age.

We obtained the PM_2.5_ data from the U.S. EPA’s AirData database (http://www.epa.gov/oar/data/), which included 1,006 monitors for the period 2000–2005. We calculated mean annual PM_2.5_ values for the study period for all 4,568 ZIP codes with centroids within 6 miles of a monitor with > 10 months of data per year. If three or more observations were available for a month, we considered this amount of data sufficient because PM concentration was measured every sixth day at many locations. Because the focus of this study was to estimate the effect of long-term exposure to PM_2.5_, we used a ZIP code 6-year average of PM_2.5_ as a measure of the long-term exposure to PM _2.5_ for an individual living within a ZIP code both during the 6 years of follow-up and for some time before cohort enrollment. We omitted the 1999 PM_2.5_ data because this was the initial year of the U.S. EPA monitoring program and coverage was limited.

**Figure 1:**
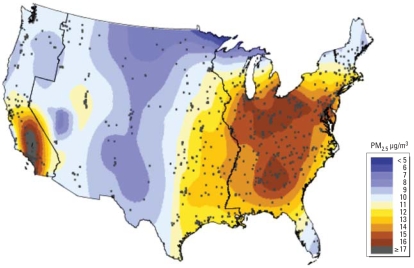
Map of spatially smoothed averages of PM_2.5_ during the study period 2000–2005. The map also indicates 4,568 ZIP code centroid locations (black circles) and western, central, and eastern U.S. regions.

An advantage of MCAPS is that it comprises persons ≥ 65 years of age from nearly all of the major urban ZIP codes in the United States, and large numbers of deaths are reported within each age stratum and region. We have therefore estimated the age- and region-specific relative risks of chronic PM_2.5_ exposure for *a*) the eastern region of the United States, with 2,938 ZIP codes in 421 counties; *b*) the central region, with 990 ZIP codes within 185 counties located between the Mississippi River and the Sierra Nevada range; and *c*) the western United States, with 640 ZIP codes within 62 counties extending from Washington State to Southern California. Figure 1 shows the location of the 4,568 ZIP code centroids, the three geographic regions, and the spatially smoothed levels of the 6-year average PM_2.5_. These spatially smoothed PM_2.5_ levels should be interpreted with caution because of the sparseness of monitors in some areas.

We conducted the analyses separately within each of these three geographic regions and for three distinct age strata: 65–74, 75–84, and ≥ 85 years of age. We also stratified initial analyses by sex and by the ZIP codes that were above and below the national median for education and income variables. Because the estimated effects for men and women and for high- and low-SES subgroups were very similar, we did not stratify the analyses reported here by sex or SES. The results of these stratified analyses are available in the Supplemental Material, Table 1 (online at http://www.ehponline.org/members/2008/11449/suppl.pdf).

In estimating the effect on mortality of PM or other air pollutants, previous cohort studies and this new study rely entirely on cross-sectional comparisons of covariate-adjusted mortality rates across geographic locations with different PM levels, because PM is not time varying in the analyses. Previous studies have accounted for potential confounding by *a*) individual-level lifestyle factors, including age and smoking, and *b*) area-level characteristics such as county-level SES. The MCAPS provides individual-level age, sex, and race data but not data on lifestyle factors. To account for SES at the ZIP code level, we used age-specific SES variables from the 2000 U.S. Census. After preliminary analysis, we selected five SES variables at the ZIP code level from the U.S. Census Bureau’s Summary File 3. We restricted the analysis to those enrollees who report ZIP codes to CMS that correspond to ZIP code tabulation areas recognized by the U.S. Census Bureau. We selected two education variables, percentage of the population with a high school diploma and the percentage with a higher education degree, along with two household income measures, percentage of households living below the poverty level and median household income, as well as percentage unemployed. To create a univariate measure of SES by which to stratify the analysis, we averaged the ranks of the five SES variables for each county.

Previous cohort studies have found little effect of adjusting for self-reported smoking status (Krewski et al. 2000). Area-level differences in cigarette smoking, however, could potentially confound the association between PM_2.5_ and mortality. Because the MCAPS data have neither individual- nor area-level smoking information, we used data from the NCHS to calculate the standardized mortality ratio (SMR) for chronic obstructive pulmonary disease (COPD) for the period 1993–2002, adjusted for age, race, and sex for each county. Because the vast majority of deaths from COPD in the United States are attributable to smoking (U.S. Department of Health and Human Services 2004), we used the SMR for COPD as a surrogate indicator of the long-term smoking pattern of its residents. We included the county-level COPD SMR in the regression model, assigning the county value to all ZIP codes within a county.

For exposure, reliance on ZIP code–level rather than county-level PM concentration is a strength, but person-level covariate information is unavailable. To assess the potential consequences of imperfect control for confounding variables, we estimated the main models with three levels of adjustment: no control for ZIP code–level confounders, control for ZIP code–level SES variables, and control for ZIP code–level SES and county-level COPD SMRs.

Within each age stratum, we estimated the following log-linear regression models (McCullagh and Nelder 1989):


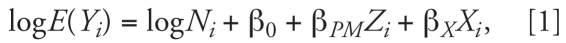


where *Y_i_*, *N_i_*, *Z_i_*, and *X_i_* are the number of deaths, number of person-years at risk, PM_2.5_, and SES and COPD SMR for ZIP code *i*. The parameter β_PM_ denotes the log relative risk of mortality associated with a 1-μg/m^3^ difference in average PM_2.5_ comparing ZIP codes that are otherwise similar with respect to SES and COPD SMR.

We report results for each region by age stratum and aggregated over the three age groups. To obtain the aggregated value, we fit a single log-linear regression with a common PM effect across the strata. We use generalized estimating equations (Diggle et al. 2002) to account for the correlation among age groups from the same ZIP code.

We carried out all analyses with the statistical programs R (R Development Core Team, Vienna, Austria) and SAS (version 9.1; SAS Institute Inc., Cary, NC). Programs are available from the authors.

## Results

Table 1 presents the total number of ZIP codes, PM_2.5_ monitors, study population, person-years of follow-up, number of deaths, and crude death rates for the eastern, central, and western regions. The study population comprises 19.1 million persons followed for a total of 92.6 million person-years or an average of 4.8 years per person. An individual can contribute person-time to two age categories, so the age-specific numbers of people do not add to the total size of the population. There were 4.88 million deaths, for a crude mortality rate of 52.6 deaths per 1,000 person-years. The crude mortality in the western region was lower by roughly 4 deaths per 1,000 person-years compared with the other two regions, reflecting its younger population.

Table 2 presents the median and inter-quartile range of the ZIP code values of average PM_2.5_ for 2000–2005, five SES variables, and COPD SMRs by region. A scatterplot matrix [see Supplemental Material, Figure 1 (online at http://www.ehponline.org/members/2008/11449/suppl.pdf)] provides an *X–Y* graph for each variable against each other variable. The proportions have been transformed to the log odds (logit) scale {log[*p*/(1 – *p*)]} to allow them to range over the whole real line rather than in (0,1); we show SES variables on a log scale to linearize their associations with mortality and to reduce the impact of a few ZIP codes with larger average incomes. The bottom row of Table 2 shows the pattern of pairwise associations between the logit of mortality and each of the covariates or PM_2.5_. As expected, mortality has a strong negative association with each of the SES variables and a positive association with COPD SMRs.

Table 3 presents the estimated relative risks stratified by region. The MCAPS data provide evidence of an association between long-term exposure to PM_2.5_ and mortality in the eastern and central regions. For the eastern ZIP codes, we found that a ZIP code with 10 μg/m^3^ higher long-term average of PM_2.5_ compared with another ZIP code with comparable age distribution, SES, and COPD SMR has a 6.8% higher mortality [95% confidence interval (CI), 4.9–8.7]. For the central ZIP codes, a 10-μg/m^3^ increase in the long-term average of PM_2.5_ is associated with a 13.2% increase in mortality (95% CI, 9.5–16.9). For the ZIP codes in the western region, the association between PM_2.5_ and mortality does not achieve statistical significance. In the eastern region, adjustment for SES and COPD SMR substantially attenuates the association from 15.5% down to 6.8% per 10 μg/m^3^ increase.

Table 4 presents the estimated region-specific log relative risks of death for each of the three age groups. In the western region, there is no evidence of an association for any of the three age groups. In the eastern and central regions, the largest effect is for the youngest group, 65- to 74-year-olds (11.4% and 20.4% per 10-μg/m^3^ increase, respectively). The effects are smaller for the 75- to 84-year-olds and close to 0 for the oldest group, those ≥ 85 years of age. Hence, there is no evidence of a PM effect for persons ≥ 85 years of age in any of the three regions.

We verified the sensitivity of the inferences to the specific choice of model used to control for SES and COPD mortality rate, and the results are qualitatively robust, as shown in Table 3. We also conducted analyses stratified by sex and by ZIP codes above and below the national median for education and income variables. We found that the estimated effects for men and women and for high- and low-SES subgroups were very similar. We report the results of these analyses in the Supplemental Material, Table 1 (online at http://www.ehponline.org/members/2008/11449/suppl.pdf).

## Discussion

In this article we present results from MCAPS, the largest study of potential health effects of chronic exposure to air pollution on morbidity and mortality to date, with 4.88 million deaths during more than 92 million person-years of follow-up. In comparison, a total of 20,765 deaths in the subcohort of the American Cancer Society (Krewski et al. 2000) were included in the analyses of air pollution and mortality, less than one-tenth the number in our study. However, CPS-II had an extensive set of individual-level risk factors. Given the availability of 1,006 air pollution monitors and mortality data from 4,568 ZIP codes within 668 urban counties, we have stratified the analyses geographically, choosing strata that broadly reflected differing source mixes and background disease patterns. This stratification also controls for potential confounders that vary on broad geographic scales.

Our estimated associations between long-term exposure to PM_2.5_ and mortality for the eastern and central ZIP codes give results qualitatively similar to those previously published from the Six Cities Study (Dockery et al. 1993) and CPS-II (Pope et al. 2002). We previously reported the comparability of MCAPS estimates to estimates from these studies, with the MCAPS cohort restricted to the 110 and the 6 counties corresponding to the 50 metropolitan areas and the 6 counties included in CPS-II and the Six Cities Study, respectively (Eftim et al. 2008). The MCAPS relative risk estimates, based on the 4,568 ZIP codes, are 11.4% and 20.4% per 10-μg/m^3^ increase in the eastern and central regions (95% CI, 8.8–14.1% and 15.0–25.8%, respectively) for the youngest age group, compared with the Six Cities Study and CPS-II values of 15.3% and 12.4%, respectively. Although the MCAPS data lack individual-level risk factor information, the MCAPS results were not qualitatively changed with inclusion of ZIP code–level or county-level SES indicators and the COPD SMR in the log-linear regression model (Tables 3 and 4). The size of the positive estimates does change with control for SES and COPD SMRs in the eastern region.

In MCAPS, we found compelling evidence of differing PM relative risks by age and geographic location. MCAPS estimates of the PM relative risk decline with increasing age category (Table 4), with no evidence of an association among persons ≥ 85 years of age. This decline may reflect the many competing causes of death for which the hazard of death increases with age. If only a subset of the competing causes is influenced by exposure to PM, then the PM-associated relative risk will reduce with age.

The MCAPS results indicate that the estimated positive association between PM_2.5_ concentration and mortality derives entirely from the eastern and central United States. A provocative finding is that the MCAPS data show no evidence of a positive association between ZIP code–level PM_2.5_ and mortality rates for the 640 urban ZIP codes in the western region. This lack of association is largely because the Los Angeles basin counties (California) have higher PM levels than other West Coast urban centers, but not higher adjusted mortality rates.

Recent multisite time-series studies of the same Medicare data also suggest that the effects of airborne PM vary by region and season. In a study of cause-specific cardiovascular and respiratory hospital admissions and daily PM_2.5_ levels in Medicare enrollees, Dominici et al. (2006) found strong regional patterns of effect across the 204 U.S. counties included during the period 1999–2002. Effect estimates for most of the cardiovascular causes were statistically significant in the eastern United States, but not in the western United States. These results were confirmed by a recent study (Bell ML et al., in press) that covered the period 2000–2005.

Previous studies of the mortality effects of chronic PM exposure or surrogates for populations in the western region have reported a range of relative risks. Most recently, Jerrett et al. (2005) investigated the PM–mortality association in a subset of the CPS-II cohort living in Los Angeles. They estimated an 11% increase in mortality per 10-μg/m^3^ increase PM_2.5_ (95% CI, –1% to 25%), using a chronic PM exposure interpolated with a statistical model of measured PM, traffic patterns, and proximity to freeways. Abbey et al. (1999) reported a follow-up analysis of data from the Adventist Health Study (Hodgkin et al. 1984) of > 6,000 nonsmoking residents of three air basins in California—San Francisco, Los Angeles, and San Diego—enrolled in 1977. They found a nonsignificant increase in all-cause deaths of roughly 5% per 10-μg/m^3^ increase PM_10_ (PM with aerodynamic diameter < 10 μm) in males and no effect in females. They reported a statistically significant association in respiratory deaths with the fraction of days > 100 μg/m^3^ PM_10_ for both sexes. Enstrom (2005) tracked mortality from 1973 through 2002 in about 50,000 California participants in the first national cohort study carried out by the American Cancer Society. Using PM_2.5_ data for 11 counties in 1979–1983, he found no association across the full follow-up period and evidence of a small effect during the first decade of follow-up. Misclassification arising from the limited exposure data available may have biased this study toward the null.

Regional differences in effect estimates may be related to heterogeneity in the PM mixture. For example, higher PM_2.5_ sulfate levels are observed in the eastern United States and higher PM_2.5_ nitrate in the western United States. A recent analysis of the chemical composition of PM_2.5_ from 2000 to 2005 characterized seasonal and regional variation for > 50 chemical components (Bell et al. 2007); several other studies have investigated the chemical composition of PM in specific regions of the United States (Ostro et al., in press; Shen et al. 2007; Subramanian et al. 2007; Vega et al. 2007; Viana et al. 2008).

The relative risks estimated in this study might be affected by ecologic bias due to using aggregate rather than individual-level air pollution exposure and confounding factors. We estimate long-term exposure by taking averages of PM_2.5_ concentrations during the study period 2000–2005 for each of the 1,006 monitors. We then assign this monitor-specific long-term average to the ZIP code of residence of each enrollees with a centroid located within 6 miles from the monitor. Bias in a cross-sectional study such as this one can occur if the difference between average personal PM exposure in a ZIP code and the average ambient value used in this study covaries with PM levels across the region after adjusting for SES and COPD SMRs. By including only ZIP codes whose centroids were within 6 miles of a monitor as the units of analysis, we used exposure values that are, on average, geographically closer to residences and thus reduced the potential for this type of ecologic bias.

In a few cohort studies, exposures have been estimated at the individual level using models and residence location (Hoek et al. 2002; Jerrett et al. 2005). This approach can assign person-specific estimates of exposure, potentially reducing the effects of exposure measurement error, depending upon the accuracy and precision of the exposure model. No cohort studies have measured personal exposure directly because this is not feasible with current technologies.

For MCAPS, the covariate information about SES was available only at the ZIP code level. Smoking data were represented by the COPD SMR for the county of residence, because the direct data on prevalence were unavailable. With the data reported here, we cannot directly evaluate the potential for ecologic bias from these terms. However, Krewski et al. (2000) have done so for CPS-II and the Six Cities Study by comparing relative risks with and without controlling for individual-level characteristics, including smoking, exercise, education, and occupational exposures. They found little change in the PM relative risk with adjustment, suggesting that ecologic bias is negligible for those personal characteristics measured in these two cohort studies.

Despite these methodologic complexities, we have shown that a cohort can be established using Medicare participants and routine monitoring data for investigating air pollution and mortality on longer-term time frames. In our initial analyses of the MCAPS data, we confirmed the association between PM_2.5_ and mortality found in other studies but we found substantial and unexplained geographic heterogeneity in the effect of PM_2.5_ across the United States.

## Figures and Tables

**Table 1:** Numbers of ZIP codes, counties, monitoring sites, Medicare enrollees, person-years of follow-up, deaths, and crude death rates stratified by region and age group for MCAPS.

	U.S. region			
Characteristic	Eastern	Central	Western	All
ZIP codes	2,938	990	640	4,568
Counties	421	185	62	668
Monitoring sites	613	280	119	1,006
Persons (millions)	12.5	3.7	3.1	19.1
65–74 years	7.7	2.3	1.9	11.9
75–84 years	5.9	1.7	1.4	8.9
≥ 85 years	2.4	0.7	0.6	3.6
Person-years (millions)	61.1	17.2	14.4	92.6
65–74 years	30.2	8.8	7.3	46.3
75–84 years	22.6	6.2	5.3	34.1
≥ 85 years	8.2	2.2	1.9	12.3
Deaths (millions)	3.26	0.91	0.70	4.88
65–74 years	0.77	0.22	0.16	1.66
75–84 years	1.31	0.36	0.28	1.95
≥ 85 years	1.18	0.33	0.26	1.77
Crude death rate (deaths/1,000 person-years)	53.4	53.1	48.8	52.6
65–74 years	25.6	25.3	22.4	25.0
75–84 years	57.8	58.7	53.3	57.2
≥ 85 years	143.6	148.9	139.1	143.9

**Table 2:** Median (interquartile range) ZIP code–level SES values, median county-level COPD SMR, and median ZIP code–level PM_2.5_, by region in MCAPS.

	U.S. region			
Characteristic	Eastern	Central	Western	All
Percent with high school degree	50.0 (41.8–56.0)	49.6 (42.1–56.1)	44.4 (37.0–51.3)	49.3 (40.8–55.6)
Percent with higher degree	28.0 (19.7–41.9)	29.2 (18.2–43.9)	31.4 (21.0–44.7)	28.7 (19.5–43.0)
Percent in poverty	10.1 (5.5–18.5)	12.4 (6.9–20.6)	12.0 (7.0–19.3)	10.9 (6.0–19.2)
Percent unemployment	5.1 (3.5–8.3)	5.3 (3.6–8.2)	6.4 (4.5–8.9)	5.3 (3.6–8.4)
Median income (thousands US$)	40.6 (31.6–52.6)	37.2 (29.8–48.2)	43.9 (35.0–56.4)	40.4 (31.5–52.3)
COPD SMR	94.0 (84.0–108.7)	109.2 (95.2–101.9)	101.9 (100.5–115.0)	99.6 (88.6–113.3)
PM_2.5_ (μg/m3)	14.0 (12.3–15.3)	10.7 (9.8–12.2)	13.1 (10.4–18.5)	13.2 (11.1–14.9)

**Table 3:** Percentage increase (95% CI) in mortality rate per 10-μg/m^3^ increase in PM_2.5_ from the log-linear regression model and stratified by three regions, and relative risks for three levels of adjustment for demographic and socioeconomic variables.

	U.S. region		
Adjustment	Eastern (*n* = 2,938 ZIP codes)	Central (*n* = 990 ZIP codes)	Western (*n* = 640 ZIP codes)
Age	15.5 (13.0 to 18.0)	17.8 (13.3 to 22.2)	0.3 (−1.9 to 2.5)
Age + SES	10.5 (8.4 to 12.5)	8.9 (5.2 to 12.5)	−0.3 (−2.2 to 1.6)
Age + SES + COPD	6.8 (4.9 to 8.7)	13.2 (9.5 to 16.9)	−1.1 (−3.0 to 0.8)

**Table 4:** Percentage increase (95% CI) in mortality rate per 10-μg/m^3^ increase in PM_2.5_ from log-linear regression using MCAPS regional data adjusting for three levels of demographic and socioeconomic variables.

	Age group (years)			
U.S. region/adjustment	All	65–74	75–84	≥ 85
Eastern				
Age	15.5 (13.0 to 18.0)	31.1 (26.8 to 35.5)	17.6 (14.9 to 20.4)	−1.4 (−3.5 to 0.8)
Age + SES	10.5 (8.4 to 12.5)	17.3 (14.6 to 20.0)	12.4 (10.1 to 14.6)	1.4 (−0.7 to 3.5)
Age + SES + COPD	6.8 (4.9 to 8.7)	11.4 (8.8 to 14.1)	8.9 (6.8 to 11.0)	1.7 (−0.3 to 3.7)
Central				
Age	17.8 (13.3 to 22.2)	39.0 (29.7 to 48.2)	17.5 (12.7 to 22.2)	−2.1 (−5.9 to 1.6)
Age + SES	8.9 (5.2 to 12.5)	16.5 (10.9 to 22.1)	8.8 (4.6 to 13.0)	−0.7 (−4.2 to 2.8)
Age + SES + COPD	13.2 (9.5 to 16.9)	20.4 (15.0 to 25.8)	12.0 (7.6 to 16.4)	−0.3 (−4.0 to 3.3)
Western				
Age	0.3 (−1.9 to 2.5)	6.0 (2.3 to 9.6)	0.4 (−2.0 to 2.7)	−5.2 (−7.2 to 3.2)
Age + SES	−0.3 (−2.2 to 1.6)	−2.1 (−5.0 to 0.8)	0.3 (−1.8 to 2.5)	0.9 (−0.8 to 2.7)
Age + SES + COPD	−1.1 (−3.0 to 0.8)	−1.5 (−4.2 to 1.1)	−0.2 (−2.2 to 1.9)	−0.5 (−2.5 to 1.5)
